# Molecular Evolutionary Analyses of the Fusion Genes in Human Parainfluenza Virus Type 4

**DOI:** 10.3390/microorganisms12081633

**Published:** 2024-08-09

**Authors:** Fuminori Mizukoshi, Hirokazu Kimura, Satoko Sugimoto, Ryusuke Kimura, Norika Nagasawa, Yuriko Hayashi, Koichi Hashimoto, Mitsuaki Hosoya, Kazuya Shirato, Akihide Ryo

**Affiliations:** 1 Department of Virology III, National Institute of Infectious Diseases, Musashimurayama-shi 208-0011, Tokyo, Japan; ssugimo@niid.go.jp (S.S.); shirato@niid.go.jp (K.S.); aryo@niid.go.jp (A.R.); 2 Department of Health Science, Graduate School of Health Sciences, Gunma Paz University, Takasaki-shi 370-0006, Gunma, Japan; nagasawa@paz.ac.jp (N.N.); hayashi@paz.ac.jp (Y.H.); 3 Advanced Medical Science Research Center, Gunma Paz University Research Institute, Shibukawa-shi 377-0008, Gunma, Japan; 4 Department of Clinical Engineering, Faculty of Medical Technology, Gunma Paz University, Takasaki-shi 370-0006, Gunma, Japan; 5 Research Center for Biosafety, Laboratory Animal and Pathogen Bank, National Institute of Infectious Diseases, Musashimurayama-shi 208-0011, Tokyo, Japan; 6 Department of Bacteriology, Graduate School of Medicine, Gunma University, Maebashi-shi 371-8511, Gunma, Japan; m2220015@gunma-u.ac.jp; 7 Department of Pediatrics, School of Medicine, Fukushima Medical University, Fukushima-shi 960-1295, Fukushima, Japan; don@fmu.ac.jp; 8 Department of Perinatology and Pediatrics for Regional Medical Support, Fukushima Medical University, Fukushima-shi 960-1295, Fukushima, Japan; mhosoya@fmu.ac.jp

**Keywords:** human parainfluenza virus 4, molecular evolution, *fusion* gene

## Abstract

The human parainfluenza virus type 4 (HPIV4) can be classified into two distinct subtypes, 4a and 4b. The full lengths of the *fusion* gene (*F* gene) of 48 HPIV4 strains collected during the period of 1966–2022 were analyzed. Based on these gene sequences, the time-scaled evolutionary tree was constructed using Bayesian Markov chain Monte Carlo methods. A phylogenetic tree showed that the first division of the two subtypes occurred around 1823, and the most recent common ancestors of each type, 4a and 4b, existed until about 1940 and 1939, respectively. Although the mean genetic distances of all strains were relatively wide, the distances in each subtype were not wide, indicating that this gene was conserved in each subtype. The evolutionary rates of the genes were relatively low (4.41 × 10^−4^ substitutions/site/year). Moreover, conformational B-cell epitopes were predicted in the apex of the trimer fusion protein. These results suggest that HPIV4 subtypes diverged 200 years ago and the progenies further diverged and evolved.

## 1. Introduction

Human parainfluenza viruses (HPIVs), which belong to the family *Paramyxoviridae*, are classified into four types genetically: HPIV2 and HPIV4 in the genus *Rubulavirus* and HPIV1 and HPIV3 in the genus *Respirovirus*. Moreover, HPIV4 is antigenically separated into two subtypes, HPIV4a and HPIV4b. These HPIVs can infect humans as the primary host and cause clinical diseases [1,2]. Acute respiratory tract infections (ARTIs), caused by various pathogens such as viruses, bacteria, and fungi, are a major health issue in children worldwide, particularly in low-income countries [3,4]. HPIVs are globally associated with ARTIs such as the common cold, croup, bronchiolitis, and pneumonia [1,2]. However, effective vaccines and antiviral agents for these viruses are not available at present [5]. Therefore, infection with HPIVs may be a public health concern.

The prevalence of each HPIV type is universally uneven [6,7]. Of the four HPIV types, HPIV-1 and HPIV-3 are the major types and are frequently detected throughout the world [6,7]. On the other hand, HPIV4 is reported to be less prevalent than other HPIVs, possibly because HPIV4 has not been widely tested or isolated [5,6]. It has been recognized that HPIV4 causes mild or asymptomatic disease but rarely leads to severe disease [6,8,9,10,11]. Therefore, it is essential to analyze HPIV4 in order to obtain novel insights into the characteristics (such as epidemiology, genetics, and clinical implications) of all HPIV types.

These HPIVs are enveloped viruses with negative-strand RNA genomes [2]. These genomes are approximately 15 kb in length and encode eight functional proteins [2]. HPIVs have two surface glycoproteins, the fusion protein (F protein) and the hemagglutinin–neuraminidase (HN), identified as the major antigens [2]. These glycoproteins play important roles in pathogenesis and infectivity [2]. The fusion protein is responsible for the fusion of the viral envelope membrane with the plasma membrane of the host cell, whereas the HN protein functions as an attachment to the cellular receptors, such as sialic acid residues [1,2].

Neutralizing antibodies can protect against several respiratory viruses that cause ARTIs. As previously reported, the fusion proteins of all HPIV types induced neutralizing responses [12]. Moreover, the F protein of respiratory syncytial virus (RSV), which belongs to the family *Paramyxoviridae*, is an important target for therapeutic drugs [13,14]. Thus, inhibition of F protein-mediated cell fusion by antibodies and small molecule inhibitors is expected to provide potential antivirals against paramyxoviruses, such as RSV and HPIVs [15]. Therefore, it is important to analyze the F protein in detail [15].

Analysis of both nucleotide and amino acid substitutions could impact the understanding of their functions, antigenicity of epitopes, and molecular evolution. Furthermore, to design and develop effective vaccines against various infectious diseases, in silico bioinformatics tools have recently been exploited to predict and screen epitopes [16,17]. Thus, pathogen genome analysis based on various bioinformatics technologies is a powerful tool to better understand not only their molecular evolution but also their characteristics and the development of antiviral strategies. However, the molecular evolution of the HPIV4 *fusion* (*F*) gene and F protein, which are important for viral cell entry and antigenicity [1,2], has heretofore been unknown. Therefore, using bioinformatics, detailed molecular evolutionary analyses of the *F* gene in HPIV4 strains detected or isolated globally from 1966 to 2022 were performed. Moreover, the structures of F proteins detected in different years for each subtype were constructed, and their predicted conformational epitope sites were compared.

## 2. Materials and Methods

### 2.1. Strains in This Study

To analyze the molecular evolution of HPIV4 *F* gene, the complete genome sequences were downloaded from NCBI Virus [https://www.ncbi.nlm.nih.gov/labs/virus/vssi/#/] (last accessed on 15 March 2024) by searching “Human orthorubulavirus 4, taxid:2560526” as a query. Nucleotide sequences, including the full-length coding region of the *F* gene (position 5174–6805; 1632 nucleotides for HPIV4 strain: M-25, NCBI Reference Sequence: NC_021928.1), were collected. In addition, strains with an uncertain sequence (e.g., N, Y, R, and V) in *F* gene or an unclear year of collection or area were excluded. Strains with 100% identity were omitted from the dataset. Finally, 48 strains remained and were used to analyze the molecular evolution of HPIV4 *F* gene. These sequences were aligned using MAFFT version v7.520 [18]. Details of the strains used in this study are presented in Supplementary Table S1.

### 2.2. Time-Scaled Phylogenetic Analyses and Estimation of Evolutionary Rate

To evaluate the molecular evolution of the HPIV4 strains and each subtype, phylogenetic trees of the *F* gene were constructed using the Bayesian Markov chain Monte Carlo (MCMC) method in the BEAST package (v.2.7.6) [19]. First, the jModelTest2 program was used to determine the suitable substitution models [20]. Second, nested sampling [21] was used to select the best combination from the six clock models (Strict Clock, Random Local Clock, Optimized Relaxed Clock, Relaxed Clock Exponential, Relaxed Clock Log Normal, and Fast Relaxed Clock Log Normal) and the two prior tree models (Coalescent Constant Population and Coalescent Exponential Population). Based on the obtained suitable models listed in Supplementary Table S2, MCMC trees were constructed by the Beast2 software. The MCMC chains consisted of 500,000,000 steps with sampling every 1000 steps. To confirm convergence, Tracer version 1.7.2 was used to evaluate effective sample sizes (ESS), and values above 200 were accepted. After burning in the first 10% of the trees, the maximum clade credibility tree was produced by TreeAnnotator version 2.7.6 in the BEAST package. The Bayesian MCMC phylogenetic tree was illustrated by FigTree version 1.4, and the 95% highest posterior densities (HPDs) of all internal nodes were computed.

In addition, the molecular evolutionary rates were estimated using suitable models selected for each dataset, as described above. Statistical analyses were performed using an unpaired *t*-test with Welch’s correction for GraphPad Prism 7 (GraphPad Software, La Jolla, CA, USA).

### 2.3. Bayesian Skyline Plot (BSP) Analyses

BSP analyses were performed using BEAST v2.7.6 to analyze the effective population size of the HPIV4 strains and each subtype [19]. The best substitution models were selected as described above. The best of the clock model in combination with the prior tree model (Coalescent Bayesian Skyline) was selected from the models (Strict Clock, Random Local Clock, Optimized Relaxed Clock, Relaxed Clock Exponential, Relaxed Clock Log Normal, and Fast Relaxed Clock Log Normal), as described above. The obtained suitable models are listed in Supplementary Table S2. The MCMC chains were run for 500,000,000 steps with sampling every 1000 steps. The BSPs were visualized with 95% HPDs using Tracer v1.7.2 [22].

### 2.4. Phylogenetic Distance Analyses

The phylogenetic distances between the HPIV4 strains were analyzed and calculated from the Maximum Likelihood (ML) tree using the Patristic program [23]. The ML phylogenetic analysis was performed using IQ-TREE version 2.2.2.6 with Model Finder, ultrafast bootstrap test parameters, and the SH-like approximate likelihood ratio test [24]. A violin plot was constructed using Orange DATA MINING version 3.35 [25]. Statistical analyses were performed using an unpaired *t*-test for GraphPad Prism 7 (GraphPad Software, La Jolla, CA, USA).

### 2.5. Selective Pressure Analyses

The non-synonymous (*dN*) and synonymous (*dS*) substitution rates at each amino acid site were calculated to identify the selective pressure sites for the fusion protein using Datamonkey [26]. Four algorithms, Single-Likelihood Ancestor Counting (SLAC) [27], Fixed Effects Likelihood (FEL) [27], Fast Unconstrained Bayesian AppRoximation (FUBAR) [28], and the Mixed Effects Model of Evolution (MEME) [29] method, were used to identify positively selected sites. Next, three methods except MEME were used to detect negatively selected sites. The significance level was set at *p* < 0.05 for SLAC, FEL, and MEME. Evidence of selective pressure for FUBAR was supported by a posterior probability > 0.9.

### 2.6. Construction of the Three-Dimensional (3D) Structure of Fusion Proteins

To compare the fusion protein structures among subtypes, 3D structural models of the fusion protein were constructed using LocalColabFold version 1.5.3 installed on a local computer [30]. Structural models of the prefusion fusion protein of HPIV4 were constructed for representative strains from each subtype (4a: NC_021928/1966, LC706552/2018, MT118676/2018, 4b: AB543337/1968, MH828708/2015, MN306058/2019, LC706555/2022). First, the multiple sequence alignment (MSA) was generated on a local computer, using uniref30 (2302) as the uniref database, PDB100 (230517) as the template dateabase, and colabfold_envdb (202108) as the environmental sequence database. Second, for the structure prediction, these flags, “--amber”, “--templates”, and “--use-gpu-relax”, were used. The number of prediction recycles was 30. Of five prediction models created with LocalColabfold for each sequence, one best model was selected, taking into account the predicted local distance difference test (pLDDT), template modeling (TM)-score, and root mean square deviation (RMSD). Finally, these final models were visualized by UCSF ChimeraX version: 1.7.1 [31].

### 2.7. Conformational B-Cell Epitope Prediction

To assess the conformational B-cell epitopes of the constructed fusion protein models, five methods, DiscoTope 3.0 (higher confidence: 1.50, recall up to ~30%) [32], ElliPro (cutoff values of 0.5) [33], epitope3D [34], SEPPA 3.0 (cutoff values of 0.089) [35], and SEMA (cutoff values of 0.76), were used [36]. Amino acid residues predicted by four or more of these methods were regarded as conformational B-cell epitopes. These predicted B-cell epitopes were mapped and colored on each model using UCSF ChimeraX version: 1.7.1 [31]. Heat maps also were constructed with the number of methods which were predicted as epitopes, using Orange DATA MINING version 3.35 [25].

## 3. Results

### 3.1. Time-Scaled Phylogeny of the F Gene in HPIV4

**Figure 1:**
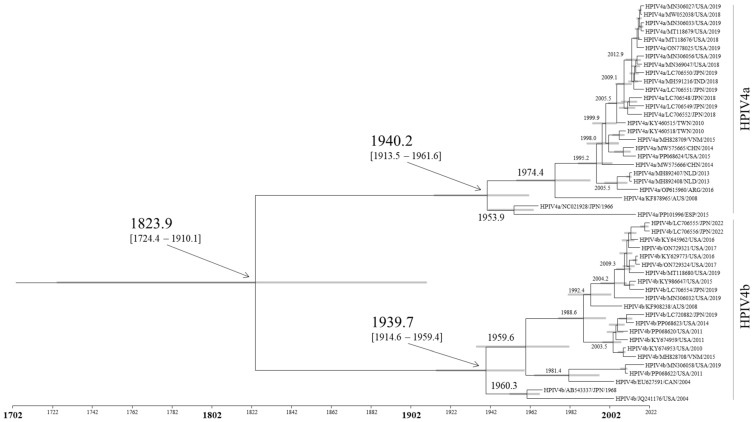
The time-scaled phylogenetic tree of the *F* gene in HPIV4 constructed by the Bayesian MCMC method. The horizontal axis represents time (years). Gray bars indicate the 95% HPD for a branched year.

A time-scale phylogenetic tree was constructed based on the full-length nucleotides of the *F* gene using the Bayesian MCMC method (Figure 1). The age at which the virus strains diverged and emerged was estimated in this analysis. A common ancestor of all HPIV4 strains appeared around 1823.9 (mean; 95% HPDs, 1724.4–1910.1). Subsequently, this HPIV4 further diverged and formed two subtypes, HPIV4a and HPIV4b. The main divergence times are shown in Figure 1. The results suggested that a common ancestor of HPIV4a and HPIV4b coincidentally diverged around 1940.2 (mean; 95% HPDs, 1913.5–1961.6) and 1939.7 (mean; 95% HPDs, 1914.6–1959.4), respectively, and evolved independently. Furthermore, HPIV4b strains diverged into subclades in 1959.6 and 1988.6. Most HPIV4 strains have been detected since 2004. However, viruses closely related to the oldest strains (HPIV4a and HPIV4b prototype strains collected in 1966 and 1968, respectively) have been detected less frequently.

### 3.2. Evolutionary Rates of the F Gene in HPIV4

The evolutionary rates of the HPIV4 *F* gene were also calculated using the Bayesian MCMC method. The speed of genetic change in a certain time period (such as per year) was estimated as the evolutionary rate. The evolutionary rate of total HPIV4 was estimated to be 4.41 × 10^−^^4^ substitutions/site/year (mean; 95% HPDs, 1.53–10.38 × 10^−^^4^ substitutions/site/year). The evolutionary rate of strains belonging to HPIV4b (mean 6.20 × 10^−^^4^; 95% HPDs, 1.75–14.32 × 10^−^^4^ substitutions/site/year) was significantly higher than that of strains belonging to HPIV4a (mean 4.27 × 10^−^^4^; 95% HPDs, 3.28–12.11 × 10^−^^4^ substitutions/site/year) (*p* < 0.0001). These results suggest that the HPIV4a and HPIV4b subtypes in the present strains evolved at different evolutionary rates, independently.

### 3.3. Phylodynamics of the F Gene in HPIV4

**Figure 2:**
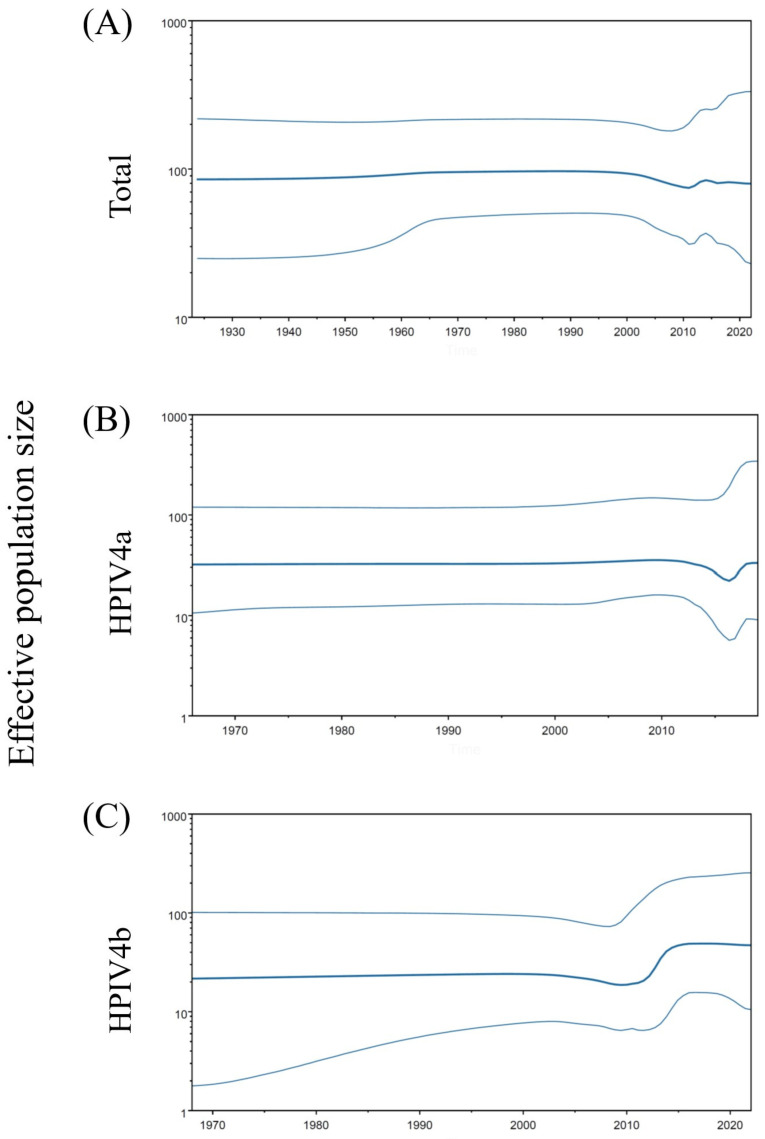
Phylodynamics of the *F* gene in total HPIV4 (**A**), HPIV4a (**B**), and HPIV4b (**C**) determined using Bayesian skyline plot analysis. The *y*-axis represents the effective population size on logarithmic scale, and the *x*-axis indicate the time in years. The thick line shows the median value over time. The intervals with the HPDs (95%) are shown by thin lines.

To assess the phylodynamics of the HPIV4 strains, time-scaled genome population sizes were calculated using the BSP method (Figure 2). The BSP method estimated past population dynamics chronologically from the dataset of nucleotide sequences and provided insight into various evolutionary processes, such as the transmission and spread of viruses. The genome population sizes of total HPIV4 and HPIV4a remained constant. However, a significant increase (from around 20 to around 50) in the genome population size of HPIV4b was observed after around 2010 and remained constant from around 2015. These results suggested that HPIV4b subtypes increased the effective population sizes, and the HPIV4b strains have adapted to humans more than HPIV4a.

### 3.4. Phylogenetic Distances of the F Gene in HPIV4

**Figure 3:**
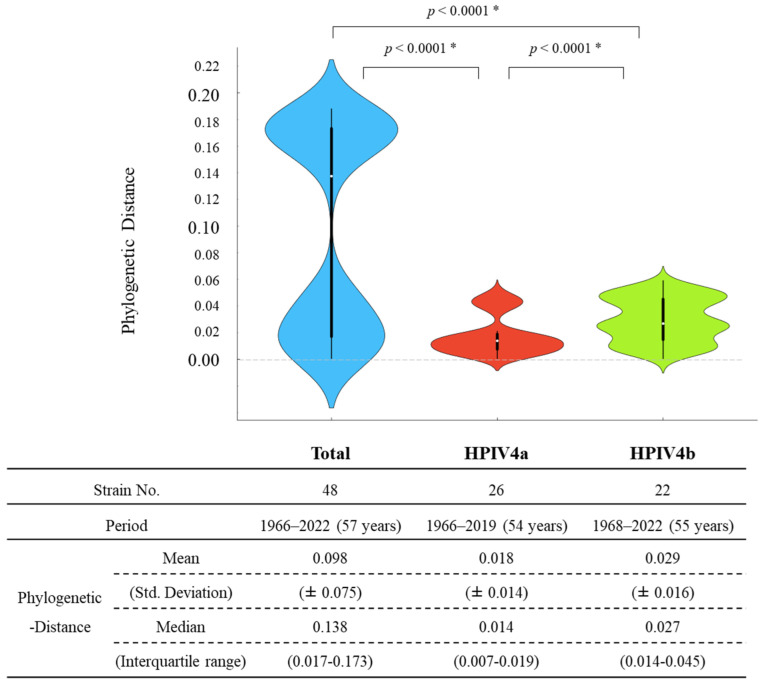
Phylogenetic distances of the *F* gene in HPIV4 illustrated by violin plots. The width of the violin plot represents kernel density, indicating the distribution shape of the data. The central box plot and white dots represent the interquartile range and the median, respectively. The whiskers from box plots represent the data intervals. The detailed statistical data are shown below the violin plots. There were significant differences in all combinations of genotypes and clusters (Unpaired *t*-test; * *p* < 0.0001).

Their phylogenetic distances and distributions were calculated to assess the genetic divergence of the HPIV4 *F* gene in the present strains. The phylogenetic distance on intra-species was estimated from branch lengths in tree files, and it summarizes the genetic change and diversity. The phylogenetic distance of the total HPIV4 *F* gene was 0.098 ± 0.075 [mean ± 1 standard deviation (SD)]. Moreover, the phylogenetic distances of strains belonging to HPIV4a and HPIV4b were 0.018 ± 0.014 (mean ± 1 SD) and 0.029 ± 0.016 (mean ± 1 SD), respectively. As shown in Figure 3, the HPIV4b *F* gene showed statistically higher genetic divergence than the HPIV4a (unpaired *t*-test, *p* < 0.0001). Detailed statistical data are shown in Figure 3. These results suggested that the *F* genes in each subtype were highly conserved.

### 3.5. Positive and Negative Selection Sites in the Fusion Protein

To determine the selective pressure against the host, positive and negative selection sites in the F protein of total HPIV4 were inferred using the Datamonkey web server. Understanding the positive and negative selection pressures can identify biologically meaningful mutation sites. The positive selection sites in the HPIV4 fusion protein were analyzed to estimate selective pressure against the host using four methods (FUBAR, FEL, MEME, and SLAC). On the F protein of total HPIV4, three residues (aa63, aa140, and aa157) were predicted as positively selected sites by only one method. No amino acid was predicted as a positive selection site by two or more methods. Thus, no amino acids were identified strongly as positive selection sites. The number of negative selection sites in the HPIV4 F protein was calculated with three methods (FUBAR, FEL, and SLAC). Thirty negative selection sites were identified using all three methods. These negative selection sites were irregularly positioned in the F protein of HPIV4. Details of these negative selection sites are shown in Supplementary Table S3.

### 3.6. Three-Dimensional Mapping of Conformational B-Cell Epitopes in the Fusion Trimer Proteins of HPIV4

**Figure 4:**
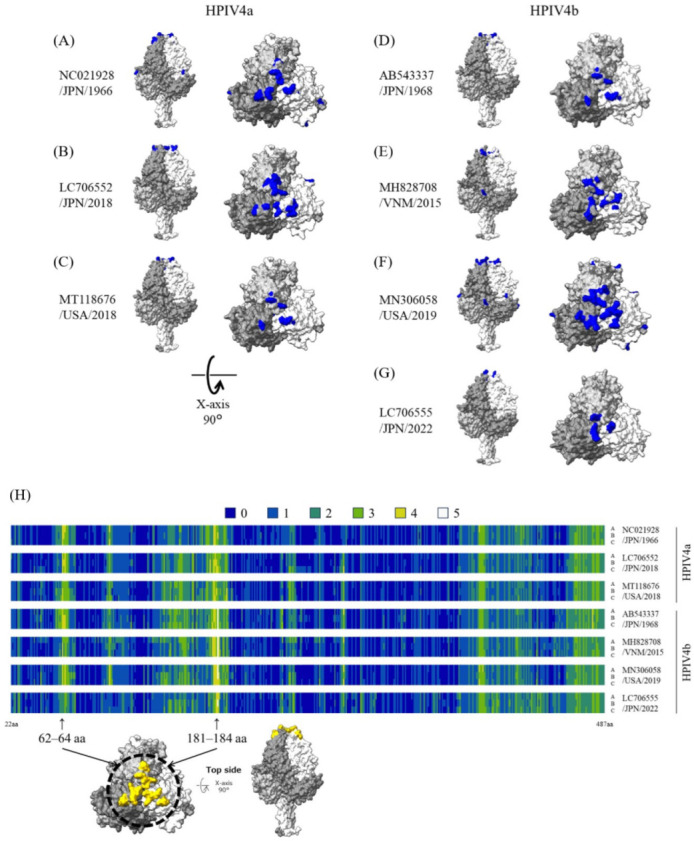
Structural models of the fusion trimer proteins and mapping of predicted conformational epitopes. Chains A, B, and C are colored dark gray, light gray, and white, respectively. The predicted conformational epitopes are shown in blue. The strains are as follows: (**A**) HPIV4a prototype strain collected in 1966 (NC021928); (**B**) HPIV4a strain collected in 2018 (LC706552); (**C**) HPIV4a strain collected in 2018 (MT118676); (**D**) HPIV4b prototype strain collected in 1968 (AB543337); (**E**) HPIV4b strain collected in 2015 (MH828708); (**F**) HPIV4b strain collected in 2019 (MN306058); (**G**) HPIV44b strain collected in 2022 (LC706555). (**H**) The sites and the number of methods which were predicted as epitopes are visualized in the heat maps. As an example, the strongly predicted epitope sites (residues aa62–64 and aa181–184) in the fusion trimer proteins are shown in yellow. Detailed information on the sites is presented in Supplementary Table S5.

The trimeric structure models of recent strains and ancient representative strains of each subtype were constructed for a computational identification of B-cell epitopes. These three or four strains of HPIV4a or HPIV4b, respectively, were selected based on low amino acid sequence homology. The predicted conformational B-cell epitopes were mapped on the fusion trimer protein (Figure 4). Details of these predicted conformational epitope sites are shown in Supplementary Table S4. In each subtype of ancient representative strains, the strongly predicted epitope sites (residues aa62–64 and aa181–184) were found at the apex of the fusion trimer proteins. However, these sites in the recent strains were no longer predicted as epitopes (Supplementary Tables S4 and S5). Among the amino acids located at these sites, residue aa63 was evaluated as the positive selection site by one method (Gln63Lys in HPIV4a and Glu63Pro in HPIV4b, respectively). Of these, HPIV4b, collected in 2022 (LC706555), especially has mutations in these sites (aa63 and 64), and these substituted amino acids were not corresponded to the epitopes (Supplementary Table S5).

## 4. Discussion

In this study, we comprehensively analyzed the evolution of the HPIV4 *F* gene/F protein using advanced and authentic bioinformatics technologies (Supplementary Table S6). The summary of the results is as follows. (1) A common ancestor of all HPIV4 strains dates back to around 1823. Subsequently, the HPIV4 viruses further diverged and formed two subtypes, HPIV4a and HPIV4b, with a relatively low evolutionary rate (Figure 1). (2) The genome population sizes of total HPIV4 and HPIV4a remained constant, while the size of HPIV4b increased after 2010 (Figure 2). (3) Overall, phylogenetic distance analyses estimated that the *F* genes of HPIV4a and HPIV4b were not genetically diverse (Figure 3). These results suggested that the HPIV4 *F* gene/F protein in each subtype was highly conserved. (4) The predicted conformational epitopes were at the apex of the F protein. To the best of our knowledge, there are no comprehensive phylogenetic analyses of the HPIV4 *F* gene/F protein so far, while most studies have focused on HPIV1 and HPIV3 [7,37,38,39,40,41,42,43]. These new findings may contribute to a better understanding of HPIV4 virology and molecular evolution.

Previous studies have reported the molecular evolution of *F* genes in HPIV1 and HPIV3 [37,40]. The most recent common ancestors of *F* genes in HPIV1 and HPIV3 emerged in 1957 and 1916, respectively, which are more recent than that in HPIV4 (1823). However, the emergence of the two HPIV4 subtypes (1940 and 1939, respectively) occurred close to that of HPIV1 and HPIV3. Moreover, the evolutionary rates of *F* genes in HPIV1 and HPIV3 were estimated as 8.504 × 10^−4^ and 9.40 × 10^−4^ substitutions/site/year, respectively [37,40], which are higher than that in HPIV4. Therefore, it is possible that the gene of HPIV4 has evolved more slowly than other HPIVs, such as HPIV1 and HPIV3.

The genome population size of HPIV4b increased in the early 2010s, while that of HPIV4a remained almost constant (Figure 2). Because there are fewer surveillance data or epidemiological reports about HPIV4, it is not possible to consider the relationship between their endemicity and fluctuations in population genome size during this period. In this study, no clinical information was provided due to using the HPIV4 sequence data from public databases. Furthermore, the number of HPIV4 detected in pediatric patients as well as adults is very small [6,8,11]. Most patients infected with HPIV4 develop mild symptoms, such as cough, fever, apnea, or asymptomatic disease [8,11]. It is possible that samples from patients with mild illness or asymptomatic cases may not be tested, resulting in low detection rates of HPIV4. Thus, this study has a limitation due to the relatively small number of strains utilized, and the paucity of clinical and epidemiological information.

The 3D models of the HPIV4 F proteins (trimer) indicated amino acid substitutions in the conformational epitopes. In this study, the apex of the HPIV4 F protein contains sites strongly predicted as epitopes. However, amino acid substitutions were found in these epitope sites of the recent strains and were not predicted as epitopes (Figure 4 and Supplementary Table S5). In particular, three amino acid substitutions were found in these sites of HPIV4b detected in 2022 (LC706555) compared to the prototype (AB543337), none of which are predicted as epitopes. Furthermore, among these amino acids, Gln63Pro was predicted as a positive selection site (however, these data were obtained from only one of the four methods). The previous studies demonstrated that antibodies bound to the apex region of the prefusion F protein neutralized HPIV3 [44,45], suggesting that the region may be an important site for defense against HPIVs infection. Therefore, mutation in this region may have been necessary for the virus to survive and infect humans. The epitopes in HPIV1 and HPIV3 F proteins, as previously reported [37,46], are compared with the present results in Supplementary Table S5. The B-cell epitopes of the HPIV1 F protein were predicted in silico in a region close to that of HPIV4 [37]. The mouse neutralizing antibody to the HPIV3 F protein also recognized the amino acid residue near the predicted B-cell epitope sites of HPIV4 [46]. Conclusively, common to all HPIVs, the apex portion of the F protein may be important for neutralizing these viruses.

These analyses, such as immunoinformatics, are practically useful tools for the development of vaccines and antiviral therapeutics [16,17]. The defensive antibodies against viruses are committed to neutralization, complement-dependent killing, phagocytosis, antibody-dependent cellular cytotoxicity, and complement-dependent cytotoxicity [47]. Among these, neutralization is considered to be the most important [47]. However, the predicted conformational B-cell epitopes may not match the neutralizing antibody binding site [40,48]. It has been reported that host immunization with the F protein of HPIV4 elicited neutralizing activity [12], but the neutralizing antibody binding sites remain unknown. Considering this limitation, it is essential to research the protective B-cell epitopes on the HPIV4 F protein, using not only bioinformatics but also in vivo or in vitro experiments (e.g., animal infection models).

As previously reported, immunization in mice with the prefusion protein of HPIVs could induce neutralizing antibodies, suggesting that the F protein is a potential vaccine candidate and antiviral target [12]. However, in this study, the number of predicted B-cell epitopes was reduced in the recent strains (Figure 4 and Supplementary Table S5). F proteins may escape the host immune system. It is possible that HPIV4 circulating in the field may evade host immunity by substituting amino acids in the apex portion of the F protein. Moreover, neutralizing antibodies to the Mumps virus, which is in the same genus, *Rubulavirus*, as HPIV4, were induced by the HN protein rather than the F protein [49]. To protect against infection by the *Paramyxoviridae* family of viruses, the immune response to the two main proteins, F protein and HN, must be understood. Thus, it is necessary to also analyze the epitopes on the HN protein on HPIV4.

The predicted B-cell epitope region in the apex portion of the F protein corresponds to site Ø of RSV belonging to the family *Paramyxoviridae*, like HPIVs. The F protein of RSV is composed of six major antigenic sites (Ø, I-V) [14,50]. Of these, antigenic site zero (Ø) is present at the apex of the trimer F protein and binds to monoclonal antibodies (MAbs) D25, AM22, AM14, and 5C4 [51,52,53]. Prefusion-specific immunization with such site Ø MAbs can protect against RSV [54]. This region is important for protection against RSV infection but is not known in HPIV4. Thus, the mutations identified in this study may be important not only for immune evasion but also for the development of antiviral drugs. Further work, such as a structural comparison and a cross-reaction with antibodies, is needed to comprehensively analyze site Ø of the F proteins of the *Paramyxoviridae* family, including HPIVs.

In conclusion, the currently detected HPIV4 viruses diverged into two subtypes (HPIV4a and 4b) around 200 years ago. An evolutionary rate of strains belonging to HPIV4b was significantly higher than that of strains belonging to HPIV4a. The HPIV4b population size increased after 2010, whereas that of HPIV4a remained constant. Moreover, the HPIV4b *F* gene showed statistically higher genetic divergence than the HPIV4a. These results suggested that HPIV4a and HPIV4b viruses have evolved independently. Furthermore, conformational B-cell epitopes were predicted in the apex of the F protein; amino acids predicted as epitopes were substituted. Together, these findings may contribute to a better understanding of HPIV4 virology and molecular evolution.

## Data Availability

The raw data supporting the conclusions of this article will be made available by the authors on request.

## Supplementary Materials

The following supporting information can be downloaded at: https://www.mdpi.com/article/10.3390/microorganisms12081633/s1, Table S1: List of HPIV4a and HPIV4b strains used in this study; Table S2: Parameters used in the Bayesian Marcov chain Monte Carlo (MCMC) analyses; Table S3: Number of amino acid residues of predicted negative selection sites in HPIV4; Table S4: Putative conformational epitopes for the fusion proteins of HPIV4; Table S5: Details of conformational B-cell epitopes at the apex of the fusion trimer proteins of HPIV4 (the number of methods which were predicted as epitopes); Table S6: Summary of methods in this study.
